# Effect of Direct Bilirubin Level on Clinical Outcome and Prognoses in Severely/Critically Ill Patients With COVID-19

**DOI:** 10.3389/fmed.2022.843505

**Published:** 2022-03-28

**Authors:** Wensen Chen, Hanting Liu, Gang Yang, Wei Wang, Qiongfang Liu, Chaolin Huang, Zhuoru Zou, Yun Liu, Guihua Zhuang, Lei Zhang

**Affiliations:** ^1^Department of Epidemiology and Biostatistics, School of Public Health, Xi’an Jiaotong University Health Science Center, Xi’an, China; ^2^Office of Infection Management, The First Affiliated Hospital of Nanjing Medical University, Nanjing, China; ^3^China–Australia Joint Research Centre for Infectious Diseases, School of Public Health, Xi’an Jiaotong University Health Science Centre, Xi’an, China; ^4^Department of Cardiology, Jiangsu Province Hospital, The First Affiliated Hospital of Nanjing Medical University, Nanjing, China; ^5^Department of Information Management, Wuhan No. 1 Hospital, Wuhan, China; ^6^Department of Infection Management, Wuhan Hankou Hospital, Wuhan, China; ^7^Center for Translational Medicine, Wuhan Jinyintan Hospital, Wuhan, China; ^8^School of Biomedical Engineering and Informatics, Nanjing Medical University, Nanjing, China; ^9^Faculty of Medicine, Nursing and Health Sciences, Central Clinical School, Monash University, Melbourne, VIC, Australia; ^10^Melbourne Sexual Health Centre, Alfred Health, Melbourne, VIC, Australia

**Keywords:** COVID-19, direct bilirubin, mortality, severely/critically disease, prognoses

## Abstract

**Objectives:**

We aimed to investigate how changes in direct bilirubin (DBiL) levels in severely/critically ill the coronavirus disease (COVID-19) patients during their first week of hospital admission affect their subsequent prognoses and mortality.

**Methods:**

We retrospectively enrolled 337 severely/critically ill COVID-19 patients with two consecutive blood tests at hospital admission and about 7 days after. Based on the trend of the two consecutive tests, we categorized patients into the normal direct bilirubin (DBiL) group (224), declined DBiL group (44) and elevated DBiL group (79).

**Results:**

The elevated DBiL group had a significantly larger proportion of critically ill patients (χ^2^-test, *p* < 0.001), a higher risk of ICU admission, respiratory failure, and shock at hospital admission (χ^2^-test, all *p* < 0.001). During hospitalization, the elevated DBiL group had significantly higher risks of shock, acute respiratory distress syndrome (ARDS), and respiratory failure (χ^2^-test, all *p* < 0.001). The same findings were observed for heart damage (χ^2^-test, *p* = 0.002) and acute renal injury (χ^2^-test, *p* = 0.009). Cox regression analysis showed the risk of mortality in the elevated DBiL group was 2.27 (95% CI: 1.50–3.43, *p* < 0.001) times higher than that in the normal DBiL group after adjusted age, initial symptom, and laboratory markers. The Receiver Operating Characteristic curve (ROC) analysis demonstrated that the second test of DBiL was consistently a better indicator of the occurrence of complications (except shock) and mortality than the first test in severely/critically ill COVID-19 patients. The area under the ROC curve (AUC) combined with two consecutive DBiL levels for respiratory failure and death was the largest.

**Conclusion:**

Elevated DBiL levels are an independent indicator for complication and mortality in COVID-19 patients. Compared with the DBiL levels at admission, DBiL levels on days 7 days of hospitalization are more advantageous in predicting the prognoses of COVID-19 in severely/critically ill patients.

## Introduction

The coronavirus disease (COVID-19) was caused by severe acute respiratory syndrome coronavirus-2 (SARS-CoV-2) infection. In late 2019, COVID-19 was first reported in Wuhan City, Hubei Province, China, when a group of hospitalized patients with pneumonia of unknown etiology was reported. Since then, the epidemic has rapidly expanded from a local outbreak to a world pandemic, as declared by the World Health Organization (WHO) on March 11, 2020 (1). Common symptoms include fever, dry cough, and shortness of breath, multiple organ dysfunction and death can occur in severe cases (2). Globally, as of November 2021, the SARS-CoV-2 pandemic has caused over 5 million deaths, reported by WHO (3). Currently, no effective antiviral regimens are yet available to cure the infection (4). The constant mutation of the virus also makes it more difficult for disease control. Early detection, effective treatment, and elucidation of the mechanisms underlying the pathogenesis of infection are urgently needed for COVID-19 patients.

SARS-CoV-2 mainly attacks the lungs, but it can also cause severe damage to the liver, kidneys, intestines, heart and the central nervous system via the ubiquitous distribution of the viral entry receptor Angiotensin-converting enzyme 2 (ACE2) (5, 6). Accumulating evidence demonstrates that liver damage is associated with clinical severity and adverse outcomes in patients with COVID-19 (7, 8). This is consistent with previous findings in patients infected with two other highly pathogenic human coronavirus infections, severe acute respiratory syndrome coronavirus (SARS-CoV) and Middle East respiratory syndrome coronavirus (MERS-CoV) (9). Herta and Berg reported a dual pattern of increased liver function abnormalities in patients with severe or critical COVID-19, characterized by hepatocellular damage that results in the elevation of serum aminotransferases in early disease onset, followed by an increase in DBiL, alkaline phosphatase (ALP) and gamma-glutamyl transferase (GGT) as the disease progresses (10). These cholestasis-associated biochemistry indicators are prognostic biomarkers of disease severity in COVID-19 patients.

Bilirubin level is a well-known biomarker for monitoring liver injury. Elevated bilirubin levels have been reported in COVID-19 patients with severe or critical diseases (11, 12). There is also evidence of cholangiocyte injury due to higher ACE2 expression—a key receptor targeted by SARS-CoV-2, which leads to DBiL elevation (13). Elevated DBiL levels indicate the presence of cholestasis. A retrospective study in the US suggested that liver injury was most often cholestatic and patients with abnormal DBiL had a higher risk of intensive care unit (ICU) admission and mortality than otherwise (14). Ding et al. showed that DBil levels in deceased COVID-19 patients had substantially increased after symptom onset and were significantly higher than those in discharged patients (15). In addition, Wu et al. conducted a study of patients with sepsis and identified the prognosis was associated particularly with DBil rather than TBil (16). These suggest that DBil is a noteworthy predictor of COVID-19-related deaths. Ng et al. pointed out that it is crucial to identify the role of DBil and indirect bilirubin (IBil) in SASR-COV-2 infection, respectively (17). Previous studies have proposed a link between bilirubin levels and disease severity, but they have not explored the relationship between DBil levels and the survival and complications of COVID-19 patients (18, 19).

This retrospective study collected the clinical records from 404 severely/critically ill patients treated in three COVID-19 designated hospitals of Wuhan between December 9, 2019, and April 3, 2020. We aimed to investigate how changes in DBiL levels in severely/critically ill COVID-19 patients during their first week of hospital admission affect their subsequent prognoses and disease endpoints. The study will provide evidence to inform clinical treatment practice for severely and critically ill COVID-19 patients.

## Materials and Methods

### Study Participants

**FIGURE 1:**
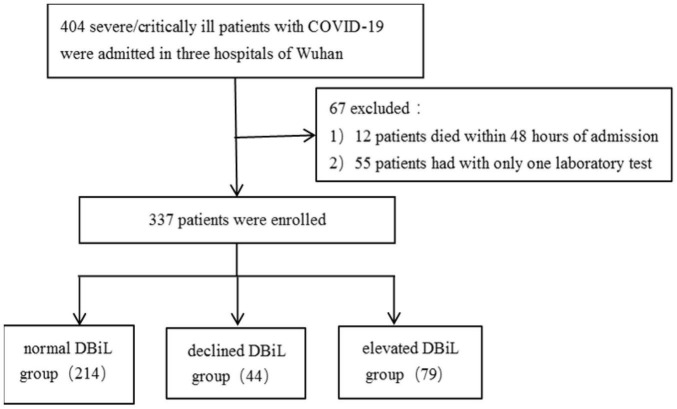
A flowchart showing the inclusion, exclusion and categorization of 404 COVID-19 severe/critically ill patients.

A cohort of 404 severely/critically ill COVID-19 patients admitted to the intensive care unit (ICU) at Wuhan Hankou Hospital, Wuhan No.1 Hospital and Wuhan Jinyintan Hospital were enrolled in this retrospective study. This retrospective study was approved by the Research Ethics Commission of Wuhan Hankou Hospital, Wuhan No.1 Hospital and Wuhan Jinyintan Hospital (HKyy202-011, 2020-SR-122, KY-2020-59.01). We excluded 12 cases that died within 48 h of admission and 55 patients who had only one laboratory test during hospitalization. Eventually, 337 patients were included in this analysis (Figure 1).

According to China’s Diagnosis and Treatment Protocol for SARS-CoV-2 (The Eighth Edition) (20), patients were diagnosed as “severe” if one or more of the following criteria were met: (1) respiratory distress (≥30 breaths/min); (2) oxygen saturation ≤93% at rest on room air; (3) arterial partial pressure of oxygen to fraction of inspired oxygen (PaO_2_/FiO_2_) ≤300 mmHg (l mmHg = 0.133 kPa). Critically ill patients were defined as those admitted to the intensive care unit (ICU), requiring mechanical ventilation or septic shock.

### Participant Categorization

Patients were categorized into three groups according to the results of the two consecutive laboratory tests of DBiL. If both laboratory test results of DBiL were ≤6.8 μmol/L, we defined it as a “normal DBiL” group. Among patients with abnormal DBiL levels, if DBiL in the second test was lower than that of the first test, we categorized them as the “declined DBiL” group. Otherwise, it is categorized as the “elevated DBiL” group (Supplementary Figure 1).

### Outcome and Clinical Indicators

A trained team of physicians and medical staff reviewed and collected the data from electronic medical records of Wuhan a Hankou Hospital, Wuhan No. 1 Hospital and Wuhan Jinyintan Hospital and checked and confirmed these records. This study’s primary outcomes were patient survival states (survival or death) and complications of the three groups during hospitalization. The demographical and clinical were collected when patients were admitted to hospitals. The indicators include age, sex, general physical measures (body temperature, resting oxygen saturation, heart rate, respiratory rate, blood pressure), symptoms at admission (fever, fatigue, cough, dyspnea, sputum, sore throat, myalgia, diarrhea, nausea, dizziness, headache, vomiting, stomachache, respiratory failure, and shock), history of complications (cardiovascular disease, cerebrovascular disease, chronic pulmonary disease, diabetes, malignancy, peptic ulcer, hemiplegia, and kidney disease).

The laboratory findings were collected from two blood tests. The first test was finished within 24 h of admission, and the second test was conducted approximately 7 days after admission. Blood test included: blood routine (leucocytes, neutrophils, lymphocytes, percentage of monocytes, red blood cell, hemoglobin, platelets) and blood biochemistry (potassium, sodium, chloride, glucose, blood urea nitrogen, serum creatinine, alanine aminotransferase, aspartate aminotransferase, TBiL, total protein, albumin, lactate dehydrogenase, creatine kinase, α- hydroxybutyrate dehydrogenase, creatine kinase isoenzyme, hypersensitive C-reactive protein, procalcitonin, prothrombin time, D-dimer).

Treatment data (medication: antiviral use, antibacterial use, corticosteroids, immunoglobulin, and traditional Chinese medicine; supportive treatment: central venous catheterization, mechanical ventilation, catheter, gastric tube, dialysis, nasal cannula, continuous renal replacement therapy, extracorporeal membrane oxygenation) and clinical outcomes (including death, discharge, and hospitalization) were also collected during the course from admission to the study endpoints. The patient’s survival time is determined by the date of death/discharge/follow-up and severe diagnosis.

### Definition of Clinical Complications

Acute respiratory distress syndrome (ARDS) was defined in accordance with the Berlin Definition (21). Heart failure was defined as a clinical syndrome characterized by typical symptoms (e.g., breathlessness, ankle swelling, and fatigue) that may be accompanied by signs (e.g., elevated jugular venous pressure, pulmonary crackles, and peripheral edema) caused by a structural and/or functional cardiac abnormality (22). Acute kidney injury (AKI) was defined was diagnosed by reference (exclusively) to serum creatinine (SCr) level, thus by an SCr increase ≥0.3 mg/dl (≥26.5 μmol/L) within 48 h, or an increase to ≥1.5-fold the baseline value, known or presumed to have developed within the prior 7 days (23). Respiratory failure was defined as a failure to maintain adequate gas exchange and is characterized by abnormalities of arterial blood gas tensions (24).

### Criteria for Discharge

Patients had to meet all the following criteria before being discharged: (1) body temperature returned to normal (<37.5°C) for three consecutive days; (2) respiratory symptoms improved substantially; (3) pulmonary imaging showed an obvious absorption of inflammation; and (4) two consecutive negative nuclei acid tests, each at least 24 h apart (25).

### Statistical Analysis

Continuous variables were reported as the median and interquartile range (IQR) and compared by the Mann-Whitney test since most laboratory data was with skewed distribution. Categorical variables were presented as counts and proportions (%) and compared by χ^2^-test or Fisher’s exact test when the data were limited. The Cox proportional hazard model was used to determine the association between different DBiL levels and the prognoses of COVID-19 patients after adjusting for potential confounders and drawing cumulative hazard function of patients in the three groups. The area under the curve (AUC) of receiver operating characteristic (ROC) was calculated to predict the disease progression and death in COVID-19 patients with elevated DBiL levels. A two-sided *P*-value less than 0.05 was considered statistically significant. We used SPSS (version 26.0) for all analyses.

## Results

### Participant Baseline Characteristics

Among the 337 participants who received two consecutive blood tests, 214 patients had normal DBiL levels; 44 patients were in the “declined DBiL” group; the remaining 79 were in the “elevated DBiL” group. In all patients, the median age was 66 years (IQR 59–75) (Table 1). The proportion of males in the declined DBiL group was the highest (79.9%; normal DBiL 56.3%; declined DBiL 73.4%, *p* = 0.002) among the three groups. Patients with elevated DBiL levels have a significantly larger proportion of critically ill patients (88.6% vs. 41.6% and 65.9%, *p* < 0.001) and a higher chance of ICU at admission (62.0% vs. 35.8% and 47.6%, *p* < 0.001) than the other two groups.

**TABLE 1:** Basic demographic characteristics, general signs, symptoms and comorbidities of 337 COVID-19 severe/critically ill patients.

	Total, *n* (%)/median (IQR)	Normal DBiL, *n* (%)/median (IQR)	Declined DBiL, *n* (%)/median (IQR)	Elevated DBiL, *n* (%)/median (IQR)	*P* value (Mann-Whitney *U*-test or χ ^2^-test)
Age	66 (59–75)	66 (57–74)	68 (59–73.75)	68 (63–75)	0.29
**Gender**					
Male	213 (63.4%)	120 (56.3%)	35 (79.6%)	58 (73.4%)	0.002*
Female	123 (36.6%)	93 (43.7%)	9 (20.4%)	21 (26.6%)	
**General signs at admission**					
Temperature	36.6 (36.4–37)	36.6 (36.4–37)	36.8 (36.5–37.5)	36.5 (36.3–37)	0.059
Respiratory rate	22 (20–26)	21 (20–25)	22 (20–30)	24 (20–31)	<0.001*
Resting oxygen saturation	92 (86.75–93)	93 (89–93)	89 (83.25–93)	91 (84–94)	0.029*
Pulse rate	88 (79.5–100)	88 (78–98)	89 (80.5–102)	89 (80–104)	0.123
Systolic blood pressure	127.5 (119–140)	125.5 (118–136)	123 (120–140)	130 (120–143)	0.211
Diastolic pressure	76 (70–82)	75 (70–81)	76 (70–80)	77 (70–86)	0.599
**Symptoms at hospital admission**					
Fever	267 (84.2%)	158 (80.2%)	34 (82.9%)	75 (94.9%)	0.010*
Fatigue	104 (37.5%)	65 (39.9%)	14 (38.9%)	25 (32.1%)	0.494
Dry cough	184 (63.7%)	120 (67.4%)	24 (68.6%)	40 (52.6%)	0.066
Dyspnea	95 (37.4%)	51 (34.5%)	18 (54.5%)	26 (35.6%)	0.091
Sputum	56 (22.1%)	38 (25.3%)	7 (23.3%)	11 (15.1%)	0.22
Sore throat	9 (3.8%)	5 (3.6%)	0 (0%)	4 (5.4%)	0.62
Myalgia	6 (2.5%)	4 (2.9%)	0 (0%)	2 (2.7%)	1
Diarrhea	11 (4.5%)	9 (6.3%)	1 (3.6%)	1 (1.4%)	0.302
nausea	8 (3.4%)	6 (4.4%)	2 (6.9%)	0 (0%)	0.066
Dizziness	16 (4.2%)	5 (3.6%)	3 (10.7%)	2 (2.7%)	0.076
Headaches	10 (4.2%)	5 (3.6%)	3 (10.7%)	2 (2.7%)	0.203
vomiting	8 (3.3%)	7 (5.1%)	1 (3.4%)	0 (0%)	0.153
Stomachache	2 (0.8%)	2 (1.5%)	0 (0%)	0 (0%)	0.645
**Comorbidities at admission**					
Myocardial infarction	6 (1.8%)	4 (1.9%)	1 (2.3%)	1 (1.3%)	1
Congestive heart failure	1 (0.3%)	1 (0.5%)	0 (0%)	0 (0%)	1
Peripheral vascular disease	84 (25.1%)	60 (28.3%)	9 (20.5%)	15 (19.2%)	0.214
Cerebrovascular disease	26 (7.8%)	15 (7.1%)	3 (6.8%)	8 (10.4%)	0.633
Dementia	5 (1.5%)	2 (0.9%)	1 (2.3%)	2 (2.6%)	0.301
COPD	6 (1.8%)	3 (1.4%)	0 (0%)	3 (3.8%)	0.378
Chronic lung disease	6 (1.8%)	2 (0.9%)	0 (0%)	4 (5.2%)	0.069
Peptic ulcer disease	2 (0.6%)	1 (0.5%)	0 (0%)	1 (1.3%)	0.597
Liver disease	11 (3.3%)	6 (2.8%)	3 (6.8%)	2 (2.6%)	0.347
Diabetes	53 (16%)	41 (19.4%)	4 (9.1%)	8 (10.4%)	0.073
Hemiplegia	8 (2.4%)	5 (2.4%)	0 (0%)	3 (3.9%)	0.505
Moderate and severe kidney disease	8 (2.4%)	4 (1.9%)	2 (4.5%)	2 (2.6%)	0.361
Tumors	11 (3.3%)	8 (3.8%)	0 (0%)	3 (3.9%)	0.544
Leukocythemia	1 (0.3%)	0 (0%)	1 (2.3%)	0 (0%)	0.133
Lymphoma	2 (0.6%)	1 (0.5%)	1 (2.3%)	0 (0%)	0.301

*Represents p-value < 0.05. COVID-19, the coronavirus disease (COVID-19); COPD, chronic obstructive pulmonary disease.

For clinical symptoms at admission, patients in the elevated DBiL group had the highest median respiratory rate compared with the other two groups (24 vs. 21 and 22, *p* < 0.001), while the median resting oxygen saturation in the declined DBiL group was the lowest among the three groups (89 vs. 93 and 91, *p* = 0.029) (Table 1). The proportion of fever (94.9%, 75/79), respiratory failure (67.1%, 53/79), and shock (26.6%, 21/79) in the elevated DBiL group were the highest among the three groups (*p* < 0.05). Existing peripheral vascular disease (25.1%) was the most common among all patients at admission, followed by diabetes (16%). The differences of these existing comorbidities were not significant (*p* > 0.05).

### Blood Tests

We observed significant differences in laboratory results at admission across three DBiL groups (Table 2). In all patients, the detected median levels of the levels of blood glucose, serum lactate dehydrogenase (LDH), α-hydroxybutyrate dehydrogenase (α-HBDH), hypersensitive C-reactive protein (hs-CRP), and D-dimer are above the normal range, whereas the median levels of lymphocyte count and albumin concentration are below the normal range. The median leucocyte count (11.5 × 109/L) and neutrophils count (9.7 × 109/L) in the elevated DBiL group were the highest compared with those in the declined DBiL group (8.4 × 109/L and 7.4 × 109/L) and the normal DBiL group (6.7 × 109/L and 5.6 × 109/L) (p < 0.001), while the median platelets counts (168.5 × 109/L) and the percentage of monocytes (3.5%) were the least compared with these two groups (declined DBiL group: 169 × 109/L, 3.3%; normal DBiL group:206 × 109/L, 5.3%) (*p* < 0.001). Besides, the elevated DBiL group had the highest median concentrations in LDH, α-HBDH, and hs-CRP (all *p* < 0.01). The median concentrations of blood urea nitrogen (BUN), AST, and D-dimer in Declined DBiL group were the highest among the three groups (all *p* < 0.001).

**TABLE 2:** Basic laboratory results of 337 COVID-19 severe/critically ill patients.

	The normal range	Total (*n* = 337), median (IQR)	Normal DBiL (*n* = 214), median (IQR)	Declined DBiL (*n* = 44), median (IQR)	Elevated DBiL (*n* = 79), median (IQR)	*P*-value (χ ^2^-test)
**Hematologic**						
Leucocyte count, × 10^9^/L	4–10	7.9 (5.2–12.4)	6.7 (5–11)	8.4 (5.2–13.4)	11.5 (7–13.9)	<0.001*
Neutrophil count, × 10^9^/L	1.2–6.8	6.6 (3.9–11.2)	5.6 (3.5–9.6)	7.4 (3.6–12.3)	9.7 (6.1–12.8)	<0.001*
Lymphocyte count, × 10^9^/L	0.8–4.0	0.7 (0.4–0.9)	0.7 (0.5–1.1)	0.6 (0.4–0.8)	0.6 (0.4–0.9)	0.002*
Percentage of monocytes (%)	4–10	4.6 (2.6–7)	5.3 (2.8–7.7)	3.3 (2.2–5.4)	3.5 (2.3–5)	<0.001*
Red blood cell count, × 10^9^/L	3.5–5.5	4.1 (3.6–4.5)	4.1 (3.6–4.4)	4.1 (3.5–4.5)	4.2 (3.7–4.6)	0.324
Hemoglobin, g/L	110–160	124 (110–136)	122 (110–135)	124 (104.8–139.5)	127 (111–141)	0.256
Platelet count, × 10^9^/L	100–300	191 (141–272)	206 (151.5–301)	169 (133.5–204.8)	168.5 (118.5–231)	<0.001*
**Biochemical (blood test)**						
Potassium, mmol/L	3.5–5.5	4.1 (3.7–4.5)	4.1 (3.8–4.6)	4 (3.5–4.6)	4 (3.4–4.3)	0.011*
Sodium, mmol/L	135–145	140 (138–143)	140 (138–143)	140 (137–143)	140.5 (138–143)	0.388
Chloride, mmol/L	96–108	105 (102.3–108.5)	105 (102–108)	105 (102–109)	106 (104–109)	0.112
Glucose, mmol/L	3.9–6.1	7 (5.5–9.2)	6.6 (5.5–9)	8.2 (5.8–9.6)	7.6 (5.8–10)	0.107
Blood urea nitrogen, mmol/L	1.8–7.1	6.7 (4.7–9.7)	5.7 (4.3–8.9)	8.6 (5.9–11)	7.5 (5.9–9.5)	<0.001*
Creatinine, μmol/L	44–133	72 (58.3–95.2)	71.2 (58–93)	78.5 (63.5–106.7)	72 (56–92.7)	0.147
ALT, U/L	0–40	31 (19–50)	26.1 (17–42)	40 (27.3–98.5)	36 (22–55)	<0.001*
AST, U/L	0–45	36.5 (25–58.3)	34 (23–51.8)	48 (33–68)	47 (29–68)	<0.001*
Total bilirubin, μmol/L	1.7–17.1	11.8 (8.6–16.4)	10.2 (7.5–13.1)	21.5 (15.6–32.3)	15.7 (11.1–21.4)	<0.001*
Total protein, g/L	60–80	62.6 (57.6–66.7)	62.2 (57.5–66.4)	62 (56.7–66.5)	63.3 (59.1–67.4)	0.316
Albumin, g/L	35–55	30.1 (27–33.2)	30.4 (27.7–34)	29.2 (25.7–33.2)	29.3 (26.9–32.1)	0.134
Lactate dehydrogenase, U/L	40–100	379 (285–548.8)	333.5 (256.5–458.8)	457 (334–579)	525 (366.5–700.5)	<0.001*
Creatine kinase, U/L	18–198	93 (51–175)	82 (50–172.5)	86 (48.5–183.5)	109 (54–222)	0.120
α-HBDH, U/L	90–182	336 (233–501)	278 (210.3–398.1)	370.5 (297.1–526)	482 (333–603.5)	<0.001*
Creatine kinase isoenzyme, U/L	0–18	15 (11–22)	14 (9–19)	18 (11.5–29.5)	20 (13.5–26)	<0.001*
Hypersensitive C-reactive protein, mg/L	0.5–10	54.2 (31.2–138.3)	37.3 (25.1–115.4)	66.5 (35.9–160)	93.1 (35.7–153.1)	0.002*
Procalcitonin, ng/mL	0–0.15	0.1 (0.1–0.4)	0.1 (0.1–0.2)	0.3 (0.1–1.5)	0.2 (0.1–0.9)	<0.001*
Prothrombin time, s	11–15	13.1 (11.8–14.7)	13.1 (11.7–14.6)	13.8 (12.3–15.9)	12.7 (11.9–14.5)	0.137
D-dimer, μg/mL	0–0.5	2.6 (0.7–10.2)	1.5 (0.6–7.2)	7.6 (0.9–19.1)	7.2 (1.2–26.9)	<0.001*

COVID-19, the coronavirus disease (COVID-19);BUN, blood urea nitrogen; ALT, alanine aminotransferase; AST, aspartate aminotransferase; TBiL, Total bilirubin; LDH, Lactate dehydrogenase; Cr, serum creatinine; CK, creatine kinase; α-HBDH, α- hydroxybutyrate dehydrogenase; CK-MB, creatine kinase isoenzyme; hsCRP, hypersensitive C-reactive protein. *Represents p-value < 0.05.

### Clinical Treatment and Outcome

The elevated DBiL group had the highest incidence of complications compared with the other two groups (*p* < 0.01), and the complications include shock (50%, 29/58), ARDS (53.8%,14/26), heart damage (34.6%, 27/78), acute renal injury (41%, 32/78), respiratory failure (80.8%, 21/26) (Table 3). Patients in the elevated DBilL group were more likely to have more treatment for complications, including central venous intubation (67.9%, 53/78), mechanical ventilation (83.3%, 65/78), the catheter (76.9%, 60/78), and gastric tube (72.2%, 57/79). The mortality of patients within 14 days of being diagnosed as severe patients in the elevated DBiL group reached 52.9% (9/17), which was above 3 times higher than that of the normal DBiL group. The proportion of discharged (11.7%, 25/214) and hospitalized (27.1%, 58/214) patients in the normal DBiL group was the highest in the three groups, while the elevated DBiL group has the highest mortality (88.6%, 70/79) (*p* < 0.001).

**TABLE 3:** Treatment, comorbidities and prognosis of 337 COVID-19 severe/critically ill patients.

	Total, *n* (%)	Normal DBiL, *n* (%)	Declined DBiL, *n* (%)	Elevated DBiL, *n* (%)	*P*-value (χ ^2^-test)
**Treatment**					
Antibiotic therapy	320 (95.5%)	201 (94.4%)	43 (97.7%)	76 (97.4%)	0.537
Corticosteroids	206 (61.7%)	130 (61.3%)	29 (65.9%)	47 (60.3%)	0.814
Antiviral therapy	244 (72.4%)	166 (77.6%)	27 (61.4%)	51 (64.6%)	0.019*
Immunoglobulin	181 (54.5%)	112 (52.8%)	28 (63.6%)	41 (53.9%)	0.421
Traditional Chinese medicine	123 (36.7%)	99 (46.3%)	10 (23.3%)	14 (17.9%)	<0.001*
Central venous catheterization	144 (42.9%)	69 (32.2%)	22 (50%)	53 (67.9%)	<0.001*
Mechanical ventilation	183 (55%)	95 (44.8%)	23 (53.5%)	65 (83.3%)	<0.001*
Gastric tube	145 (43%)	66 (30.8%)	22 (50%)	57 (72.2%)	<0.001*
Dialysis	35 (10.4%)	23 (10.7%)	2 (4.5%)	10 (12.8%)	0.344
ECMO	2 (1%)	2 (1.9%)	0 (0%)	0 (0%)	0.429
**Comorbidities**					
Shock	83 (28.2%)	42 (21.1%)	12 (32.4%)	29 (50%)	<0.001*
ARDS	45 (22.7%)	24 (16.2%)	7 (29.2%)	14 (53.8%)	<0.001*
Heart damage	87 (25.9%)	42 (19.6%)	18 (40.9%)	27 (34.6%)	0.002*
Acute renal injury	94 (28%)	49 (22.9%)	13 (29.5%)	32 (41%)	0.009*
Respiratory failure	62 (31.3%)	35 (23.6%)	6 (25%)	21 (80.8%)	<0.001*
**Prognosis**					
In-hospital death (within 14 days of being diagnosed as severe patients)	29 (21.3%)	15 (14.6%)	5 (31.3%)	9 (52.9%)	0.001*
Discharged	32 (9.5%)	25 (11.7%)	3(6.8%)	4 (5.1%)	<0.001*
Hospitalized	74 (22%)	58 (27.1%)	11(25%)	5 (6.3%)	
In-hospital death	231 (68.5%)	131 (61.2%)	30 (68.2%)	70 (88.6%)	

*Represents p-value < 0.05. COVID-19, the coronavirus disease (COVID-19); ECMO, extracorporeal membrane oxygenation; ARDS, acute respiratory distress syndrome.

### Association of Changes in Direct Bilirubin Levels With Adverse Outcomes of COVID-19 Severely/Critically Ill Patients

**FIGURE 2:**
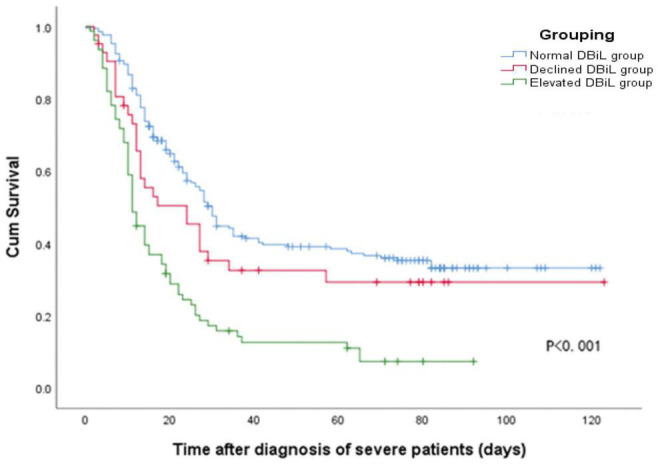
Kaplan-Meier curve shows the cumulative survival rate of 337 COVID-19 patients in different DBiL groups after being diagnosed as severely/critically ill cases.

Multivariate Cox regression analysis (Table 4, Model 3) demonstrated that the risk of mortality in the elevated DBiL group was 2.27 (95% CI: 1.50–3.43) times higher than that in the normal DBiL group after adjusted age, initial symptom, and laboratory markers (*p* < 0.001). Declined DBiL group did not show any significant difference (AHR: 1.18, 95%CI: 0.68–2.02). Similarly, the Kaplan-Meier curve shows the survival rate of COVID-19 patients in the elevated DBiL group was the lowest compared with the other two groups (*p* < 0.05) (Figure 2). ROC analysis demonstrated that the second test of DBiL measured after 7 days of hospitalization was consistently a better indicator of the occurrence of complications (except shock) and mortality than the first test measured within 24 h of admission in severe/critical COVID-19 patients (Supplementary Figure 2). The area under the ROC curve (AUC) combined with two consecutive DBiL levels for respiratory failure and death was the largest.

**TABLE 4:** Cox proportional hazards regression for death among 337 COVID-19 severe/critically ill patients with various DBiL levels.

Variable		Multivariate Analysis						
		
	Univariate Analysis		Model1*^a^*		Model2*^b^*		Model3*^c^*	
				
	HR (95%)	*P*	AHR (95%CI)	*P*	AHR (95%CI)	*P*	AHR (95%CI)	*P*

Normal DBiL	Ref		Ref		Ref		Ref	
Declined DBiL	1.35 (0.90–2.03)	0.153	1.30 (0.86–1.96)	0.211	1.39 (0.89–2.23)	0.17	1.18 (0.68–2.02)	0.56
Elevated DBiL	2.59 (1.93–3.49)	<0.001*	2.52 (1.87–3.39)	<0.001*	1.80 (1.29–2.49)	< 0.001*	2.27 (1.50–3.43)	< 0.001*

*Represents p-value < 0.05. DBiL, direct bilirubin; AHR, adjusted hazard ratio; CI: confidence interval.

*^a^Adjusted for age.*

*^b^Additionally adjusted for the resting oxygen saturation, respiratory failure, shock, cough, sputum, sore throat and vomiting.*

*^c^Additionally adjusted for white blood cells count, neutrophil count, lymphocyte count, percentage of monocytes, glucose, blood urea nitrogen, aspartate aminotransferase, total bilirubin, albumin, lactate dehydrogenase, creatine kinase isoenzyme, procalcitonin, prothrombin time, D-dimer and the National Early Warning Score.*

## Discussion

Our study investigated the association between changes in DBiL levels and COVID-19 disease progression based on 337 severely/critically ill COVID-19 patients. We found that 88.6% of patients in the elevated DBiL group died, compared with 68.5% in the normal DBiL group. Patients in the elevated DBiL group demonstrate a higher risk of mortality. This is consistent with previous findings that COVID-19 patients with abnormal DBiL levels have higher mortality (15–27). Patients with elevated DBiL levels are also more prone to respiratory failure and shock at admission and complications during hospitalization. These complications may be associated with liver dysfunction in the production of albumin, acute reactants, and coagulation factors, leading to multi-system manifestations of COVID-19, such as ARDS, coagulation, and multiple organ failure (5).

Our study indicates that a high DBiL level may reflect a severe level of liver injury among severely/critically ill COVID-19 patients. In comparison, a previous study has reported that liver injury and failure are common complications in critically ill COVID-19 patients, leading to increased COVID-19 mortality (5). Our finding is consistent with the finding that patients with severe liver dysfunction have higher bilirubin levels than otherwise. Bilirubin is derived from the catabolism of heme (predominantly hemoglobin-heme) (28); once formed, bilirubin is transported in the blood circulation as a reversible complex with serum albumin. It is subsequently absorbed into the liver where it is transformed into three different glucuronide derivatives by a specific glucuronosyltransferase enzyme (29). The glucuronide derivatives, too polar to cross the canalicular membrane by diffusion, are transported into bile by the canalicular ATP-dependent transport protein MRP2 (30). Bilirubin is an absolute requirement for glucuronidation for efficient excretion. DBiL is formed in a variety of cholestatic illnesses when the mechanism of biliary excretion of bilirubin glucuronides is impaired. Zhao et al. and Yang et al. used human liver ductal organoids to study SARS-CoV-2 infection and virus-induced tissue damage *in vitro* (31, 32). Their studies suggest that SARS-CoV-2 infection impairs the barrier and bile acid transporting functions of cholangiocytes through modulating the expression of genes involved in tight junction formation and bile acid transportation. These findings may explain the increase of DBiL in patients with COVID-19. Horvitz et al. have also pointed out that cholestasis is usually an early symptom in life-threatening conditions and a major risk factor for complications and mortality in ICU (33). This may explain the high mortality among patients in the elevated DBiL group in our study.

Our study used the ROC curve to confirm the relationship between DBiL and COVID-19. Compared with the DBiL levels at admission, DBiL level on day 7 after hospitalization is more advantageous in predicting the prognoses of COVID-19 in severely/critically ill patients. The AUC of DBiL levels of two consecutive tests for respiratory failure and death is greater than that of any single test. Liang et al. and Zhang et al. also indicated that DBiL can be used as an indicator of disease progression and prognosis in patients with COVID-19 (34, 35). In another study, Ding et al. reported that DBiL level increased gradually during the hospitalization of COVID-19 patients who died and reached the highest level before death (15). Those findings suggest that it is necessary to regularly monitor DBiL levels of COVID-19 patients during hospitalization, and repeated DBiL test results are advantageous in predicting COVID-19 prognosis.

This study has several limitations. First, only 337 patients with COVID-19 infection who came from Wuhan city were included; confirmed but only one DBiL laboratory test case was ruled out in the analyses. It would be better to include as many patients as possible in other cities to achieve a more comprehensive understanding of the association between COVID-19 and DBiL levels. Second, patients in this study were severe or critical cases, which may not be representative of the real-world situation where most COVID-19 cases are mild or moderate. Third, the lack of radiological data makes it impossible to integrate it into the analysis. Fourth, this is a retrospective study. Cohort studies or large sample case-control studies are needed to confirm further the association between changes in DBiL levels and the disease progression of COVID-19.

## Conclusion

Conclusively, our study report that both risks of complications and mortality are significantly higher in the elevated DBiL group than the normal DBiL group and the declined DBiL group. DBiL level may be an independent predicting indicator for COVID-19 complications and mortality. Compared with the DBiL levels at admission, DBiL level on day 7 after hospitalization is more advantageous in predicting the prognoses of COVID-19 in severely/critically ill patients.

## Data Availability Statement

The raw data supporting the conclusions of this article will be made available by the authors, without undue reservation.

## Ethics Statement

The studies involving human participants were reviewed and approved by the Wuhan Hankou Hospital, Hubei, China (HKyy202-011); Wuhan No. 1 Hospital, Hubei, China (2020-SR-122); Wuhan Jinyintan Hospital, Hubei, China (KY-2020-59.01). Written informed consent for participation was not required for this study in accordance with the national legislation and the institutional requirements. Written informed consent was not obtained from the individual(s) for the publication of any potentially identifiable images or data included in this article.

## Author Contributions

WC, HL, ZZ, and LZ contributed to the conception and design of the study. HL performed the statistical analysis and wrote the first draft of the manuscript. WC, GY, WW, QL, and CH contributed to data acquisition. HL, WC, LZ, and GZ contributed to manuscript revision. All authors contributed to data interpretation and approved the final version.

## Conflict of Interest

The authors declare that the research was conducted in the absence of any commercial or financial relationships that could be construed as a potential conflict of interest.

## Publisher’s Note

All claims expressed in this article are solely those of the authors and do not necessarily represent those of their affiliated organizations, or those of the publisher, the editors and the reviewers. Any product that may be evaluated in this article, or claim that may be made by its manufacturer, is not guaranteed or endorsed by the publisher.
