# Supplementary material for: Effect of Direct Bilirubin Level on Clinical Outcome and Prognoses in Severely/Critically Ill Patients With COVID-19
Source: Front Med (Lausanne). 2022 Mar 28;9:843505. doi: 10.3389/fmed.2022.843505 (PMC8996189; doi:10.3389/fmed.2022.843505)
Supplement: Supplementary file 1 [file Data_Sheet_1.pdf]

Supplementary Appendix:

Effect of changes of direct bilirubin level on clinical outcome and prognoses in severely/critically ill patients with COVID-1

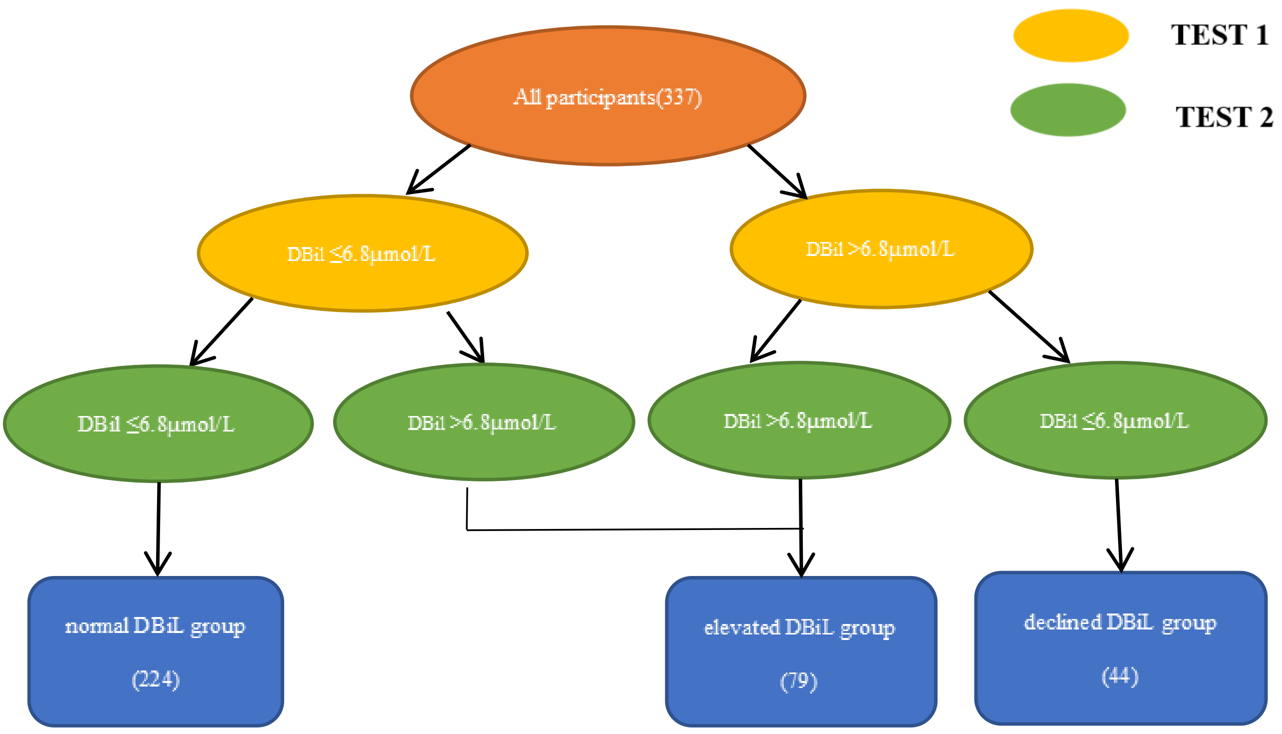

Figure S1 A flowchart showing the categorization of 337 COVID-19 severe/critically ill patients.

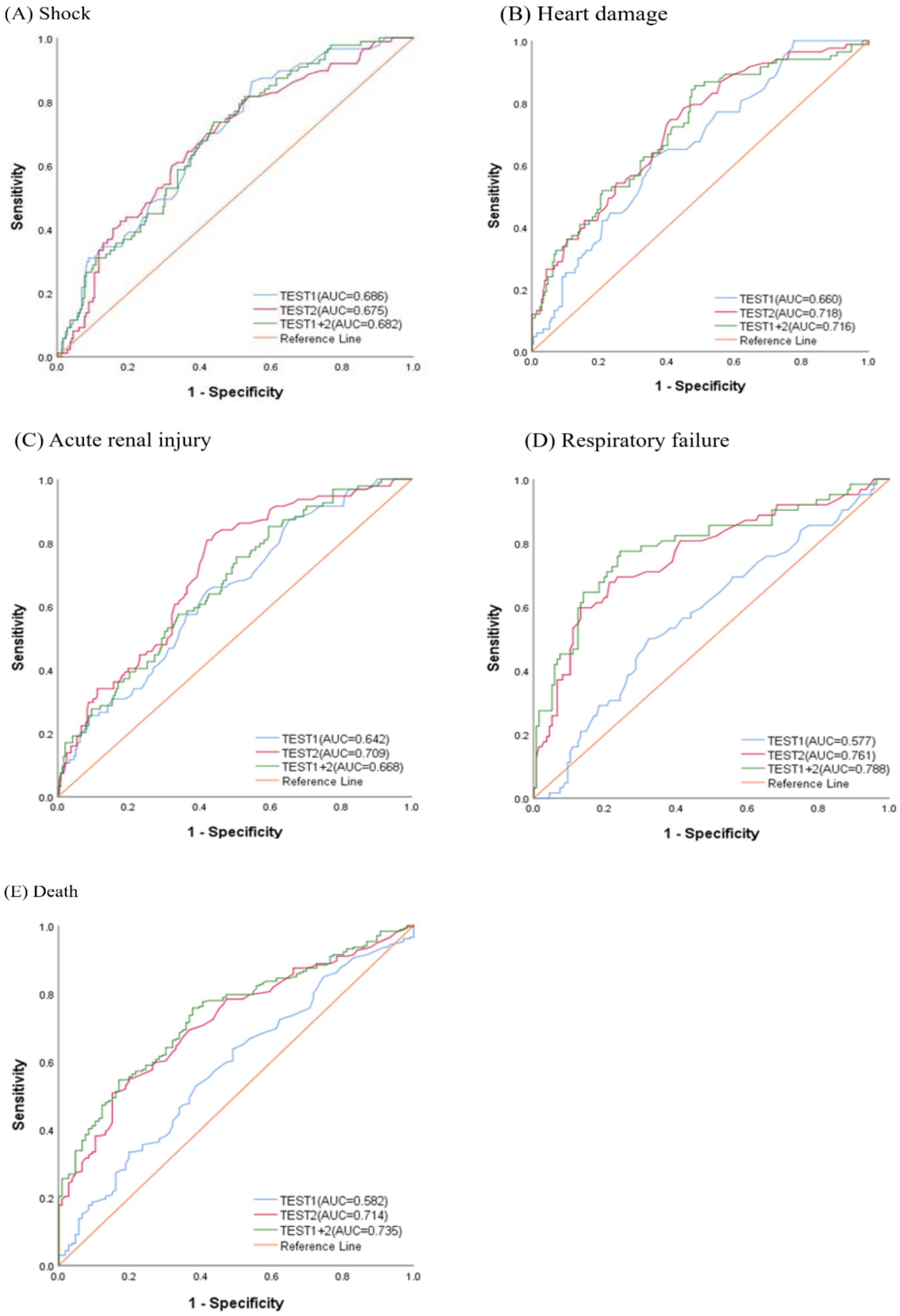

Figure S2 ROC curve of various DBiL levels and adverse outcome measured at admission (TEST1) and about seven days after admission (TEST2) among 337 COVID-19 severe/critically ill patients.

Table S1 Laboratory results about seven days after admission of 337 COVID-19 severe/critically ill patients.

	the normal range	Total, median (IQR)	Normal DBiL, median (IQR)	Declined DBiL, median (IQR)	Elevated DBiL, median (IQR)	P value (Mann-Whitney U test)
**Hematologic**						
White blood cells count, ×109/L	4-10	8.7(6-12.7)	7.9(5.5-11.8)	8.7(5.6-13.4)	11.7(8.3-16.3)	<0.001*
Neutrophil count, $\times 10^9$/L	1.2-6.8	7.7(4.6-12.1)	6.7(3.9-10.5)	7.3(4-12.5)	10.7(7.3-15.4)	<0.001*
Lymphocyte count, ×109/L	0.8-4.0	0.7(0.5-1.1)	0.8(0.5-1.2)	0.7(0.4-1.1)	0.5(0.4-0.9)	<0.001*
Percentage of monocytes (%)	4-10	4.5(2.4-7.1)	5.6(2.9-7.8)	4.2(2.4-6.7)	2.9(1.8-4.1)	<0.001*
Red blood cell count, ×109/L	3.5-5.5	3.9(3.5-4.4)	3.9(3.5-4.3)	3.9(3.4-4.5)	4(3.5-4.4)	0.626
Haemoglobin, g/L	110-160	118(106.8-133)	117.5(106.3-132)	115(104-135)	123(107-137)	0.16
Platelet count, $\times 10^9$/L	100-300	188(125-256)	216(149-269)	151(111-239)	140(98-216)	<0.001*
**Biochemical**						
Potassium, mmol/L	3.5-5.5	4(3.6-4.5)	4(3.6-4.5)	4(3.6-4.7)	4(3.6-4.6)	0.93
Sodium, mmol/L	135-145	141(138.8-144)	141(139-143)	142(138-146)	142(138.5-148)	0.168
Chloride, mmol/L	96-108	106(103-109)	106(103-109)	106(104-110)	107(104-110)	0.064
Glucose, mmol/L	3.9-6.1	7.1(5.2-10.1)	7(5.2-9.1)	6.9(5.1-9.5)	8.4(6.1-12)	0.046
Blood urea nitrogen, mmol/L	1.8-7.1	7.2(4.8-11.6)	6.5(4.3-9.5)	8(4.9-15.2)	9.5(6.8-14.8)	<0.001*
Creatinine, µmol/L	44-133	70.9(59-98.6)	69(59-87.8)	69.9(61.6-114.3)	82.8(59.6-121)	0.039
ALT, U/L	0-40	30(19-54.8)	28(17-49)	39(22-68.8)	38(24-70)	0.002
AST, U/L	0-45	32(21-48)	25(18-39.5)	38(27-56)	43(29-65)	<0.001*
Total bilirubin, µmol/L	1.7-17.1	12.8(9.3-19.1)	10.9(7.9-13.4)	15.6(11.9-24.6)	25.4(18.9-33)	<0.001*
Total protein, g/L	60-80	61.8(57.9-66.5)	61.4(57.9-66)	62.3(57.6-66.5)	62.1(58.4-67.8)	0.83
Albumin, g/L	35-55	29.1(26.8-32.3)	29.7(27-33)	28.3(25.8-31.4)	28.8(26.8-31.3)	0.123
Lactate dehydrogenase,U/L	40-100	375(248.3-545.3)	310(221-461.5)	411(230-599.5)	536.5(368.8-711)	<0.001*
Creatine kinase, U/L	18-198	82(42-168.3)	75(39-160)	82(45-140)	91(61-197)	0.136
α-HBDH, U/L	90-182	331(221.8-443.8)	284.5(200-374.4)	353(255-476)	412(314.5-612)	<0.001*
Creatine kinase isoenzyme, U/L	0-18	15(10-22.8)	13(9-19)	19(12.8-32)	17(10.8-28.3)	0.001

Hypersensitive C-reactive	0.5-10	62.3(27.7-160)	37.2(15.2-113.5)	89.3(34.8-160)	143.4(57.2-160)	<0.001*
Procalcitonin, ng /mL	0-0.15	0.2(0.1-0.8)	0.1(0.1-0.5)	0.5(0.2-4.7)	0.7(0.2-2.3)	<0.001*
Prothrombin time, s	11-15	13.5(12.2-15.2)	13.4(11.8-14.8)	14(12.6-16.1)	14.1(12.3-16.1)	0.053
D-dimer, μg/mL	0-0.5	6.5(1.7-16.9)	3.7(1-8.4)	8(4.1-39.5)	10(4.9-29.8)	<0.001*

* represents p value <0.05.

Abbreviations: BUN, blood urea nitrogen; ALT, alanine aminotransferase; AST, aspartate aminotransferase; TBiL, Total bilirubin; LDH, Lactate dehydrogenase; Cr, serum creatinine; CK, creatine kinase; α-HBDH, α- hydroxybutyrate dehydrogenase; CK-MB, creatine kinase isoenzyme; hsCRP, Hypersensitive C-reactive protein
